# Traumatic brain injury in England and Wales: prospective audit of epidemiology, complications and standardised mortality

**DOI:** 10.1136/bmjopen-2016-012197

**Published:** 2016-11-24

**Authors:** T Lawrence, A Helmy, O Bouamra, M Woodford, F Lecky, P J Hutchinson

**Affiliations:** 1 Trauma Audit and Research Network, Manchester Medical Academic Health Sciences Centre, Institute of Population Health, University of Manchester, Salford Royal Hospital, Salford, UK; 2 Division of Neurosurgery, Department of Clinical Neuroscience, University of Cambridge, Cambridge, UK; 3 Department of Neurosurgery, Addenbrooke's Hospital, Cambridge, UK; 4 Centre for Urgent and Emergency Care Research (CURE), Health Services Research Section, School of Health and Related Research (ScHARR), University of Sheffield, Sheffield, UK

**Keywords:** NEUROSURGERY, ACCIDENT & EMERGENCY MEDICINE, TRAUMA MANAGEMENT

## Abstract

**Objectives:**

To provide a comprehensive assessment of the management of traumatic brain injury (TBI) relating to epidemiology, complications and standardised mortality across specialist units.

**Design:**

The Trauma Audit and Research Network collects data prospectively on patients suffering trauma across England and Wales. We analysed all data collected on patients with TBI between April 2014 and June 2015.

**Setting:**

Data were collected on patients presenting to emergency departments across 187 hospitals including 26 with specialist neurosurgical services, incorporating factors previously identified in the Ps14 multivariate logistic regression (Ps14^n^) model multivariate TBI outcome prediction model. The frequency and timing of secondary transfer to neurosurgical centres was assessed.

**Results:**

We identified 15 820 patients with TBI presenting to neurosurgical centres directly (6258), transferred from a district hospital to a neurosurgical centre (3682) and remaining in a district general hospital (5880). The commonest mechanisms of injury were falls in the elderly and road traffic collisions in the young, which were more likely to present in coma. In severe TBI (Glasgow Coma Score (GCS) ≤8), the median time from admission to imaging with CT scan is 0.5 hours. Median time to craniotomy from admission is 2.6 hours and median time to intracranial pressure monitoring is 3 hours. The most frequently documented complication of severe TBI is bronchopneumonia in 5% of patients. Risk-adjusted W scores derived from the Ps14^n^ model indicate that no neurosurgical unit fell outside the 3 SD limits on a funnel plot.

**Conclusions:**

We provide the first comprehensive report of the management of TBI in England and Wales, including data from all neurosurgical units. These data provide transparency and suggests equity of access to high-quality TBI management provided in England and Wales.

**Strengths and limitations of this study**

- The use of registry data from all specialist units and a large number of hospitals allows a comprehensive assessment of the management of traumatic brain injury in England and Wales.
- Data from a large number of patients provides robust statistical analyses.
- Data are limited by the prespecified categories within the Trauma Audit and Research Network (TARN) data set.
- Key parameters such as Glasgow Coma Score (GCS) are collected at a single time point at admission that may not reflect the complexity of confounding factors such as resuscitation state.

## Introduction

Traumatic brain injury (TBI) is a major cause of mortality and morbidity. In England and Wales, ∼1.4 million patients per year attend hospital following head injury and it is the most common cause of death under the age of 40 years.^1^ Over the past 30 years, advances in management including the introduction of Advanced Trauma Life Support^2^ National Institute for Health and Care Excellence (NICE) guidelines^1^ and protocol-driven therapy have improved outcome^3^ and reduced mortality.^4^ Recently, Regional Trauma Networks have been implemented in England and Wales. It is recognised, however, that major gains are still needed in terms of increasing our understanding of the pathophysiology of this heterogeneous condition and defining and optimising individual treatment strategies. The largest existing TBI data sets in the literature are from the CRASH^5^ study, ∼10 000 patients within a randomised control study of corticosteroids, and IMPACT^6^ a collated data set of ∼9800 patients from eight randomised and three observational studies.

The UK national neurosurgical society, the Society of British Neurological Surgeons (SBNS), has established the Neurosurgical National Audit Programme (NNAP)^7^ the first comprehensive national audit of emergency and elective activity in an acute surgical specialty with a complex case mix, as a mechanism for driving quality improvement and maintaining high standards of clinical governance. Hospital and consultant surgeon level data have been collected and the first year of data relating to elective activity was published on 1 December 2014. The management of TBI differs from other aspects of neurosurgical care, in that it is heavily reliant on multidisciplinary care, including emergency medicine, neurointensive care, neurosurgery and rehabilitation medicine. In this way, surgeon-specific data are less useful and the aggregate performance of a whole unit is more indicative of the quality of care that is delivered. For this reason, the SBNS and the Trauma Audit and Research Network (TARN) have collaborated in order to produce detailed data on the management of several aspects of TBI management across England and Wales in over 15 000 patients.

The objective of this study was to undertake an audit of TBI in England and Wales during a 15-month epoch (April 2014 to June 2015) specifically to define the demography, mechanism of injury, arrival mode, to characterise transfers and direct admissions to neurosurgical units, length of stay, self-reported complications and outcome in terms of mortality. We specifically sought to ascertain compliance with NICE guidance and variation in mortality according to neurosurgical centre.

## Materials and methods

The information shown in this report is derived from the TARN registry, a prospective, observational registry of hospitalised major trauma patients in England and Wales. TARN has Health Research Authority (PIAGG Section 20) approval to conduct research on anonymised data. There was no patient involvement in the design or implementation of the study other than the oversight presented by the patient and public representatives on the TARN Board. Patients of all ages are eligible for entry to the TARN database if they suffer injuries leading to a hospital stay resulting in any of: 3 or more days in hospital, admission to intensive or high dependency care, interhospital transfer or death from injury. Patients aged over 65 years with an isolated neck of femur fracture or those with isolated closed limb fractures are excluded. Those who died at the incident scene and were not transported to hospital are not eligible. Currently the TARN database contains information on over 69 000 eligible major trauma patients admitted to hospitals in England and Wales over the period of the study (April 2014 to June 2015). Each patient's injuries are centrally coded and scored reproducibly by TARN coordination centre staff using the Abbreviated Injury Scale (AIS) Dictionary^8^—each injury attracts a threat to life severity code between 1 (minimal) and 6 (maximal/incompatible with life). Of these 15 820 suffered a TBI (defined as an AIS 3 or greater injury to the head). Severe TBI was defined as an initial (ie, at the time the patient was admitted and assessed in the emergency department (ED)) Glasgow Coma Score (GCS) of 8 or less in combination with an AIS 3 or greater traumatic brain injury, moderate and mild TBI were defined as GCS 9–13 and 14–15, respectively. GCS is a composite score incorporating three categorical variables: best eye opening (E), verbal (V) and motor (M) scores and is, de facto, the most widely used stratification metric for patients with TBI. Simple cross-tabulations and percentages were used to describe the study demography, injury mechanisms and features of the care pathway (endotracheal intubation, imaging with CT scan, transfer to a neurosurgical centre, surgical interventions and in-hospital complications) by severity of TBI for the whole study sample. Bias was avoided by collecting data on all available patients.

The following analysis focuses on these 15 820 patients. Some analyses use subsets of this cohort; patients admitted directly from the scene of injury and those admitted to a neurosurgical centre. As a result of relatively small numbers of patients treated exclusively at sites without neurosurgery, these are not included in the risk-adjusted outcome analysis, this group is further filtered to only include patients whose outcome is recorded on the TARN database.

Outcome, measured as mortality is considered by using a derivation of the Ps14 multivariate logistic regression^9^ (Ps14^n^) model. The Ps14^n^ model calculates a probability of survival for each patient based on their age, gender, initial GCS, Injury Severity Score (ISS) and any pre-existing medical conditions. The Ps14^n^ model adds pupillary reactivity due to its prognostic importance in head injury.^6^ ^10^ The Ps14^n^ model was generated using 33 715 cases admitted between 2010 and 2013 (inclusive) following head injury. Missing GCS and pupil reactivity values were imputed and patients with missing pre-existing medical condition data were categorised as such. The model was internally validated using bootstrap simulation. Details of the model, including coefficients and calibration information can be found in the online supplementary information.

10.1136/bmjopen-2016-012197.supp1 supplementary data

In order to compare mortality across different centres, the predicted survival rate at 30 days or discharge (whichever is earliest), derived from the probability of survival values assigned to patients admitted to a given institution is subtracted from the observed survival rate at 30 days or discharge to generate a ‘W’ score. This is then risk standardised (Ws) to allow direct comparison between units by compensating for variations in admission patterns.^11^ A positive Ws score, therefore, indicates a better than expected rate of survival. The same outcome assessment for mortality, that is, discharge or 30-day mortality, whichever is earliest, is used in the data within the online supplementary data.

## Results

**Figure 1:**
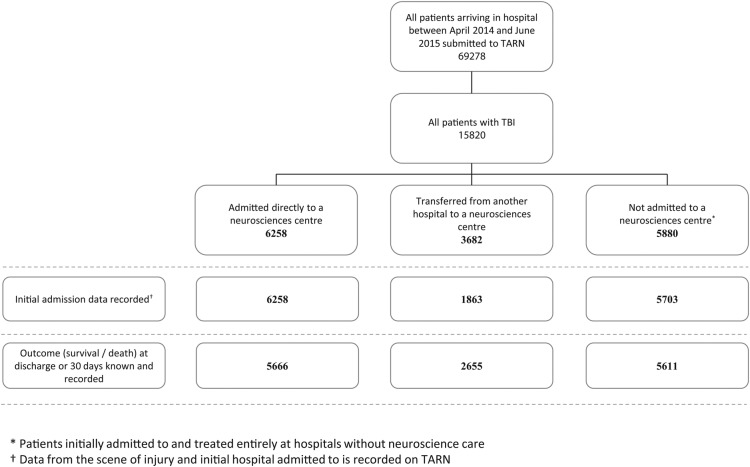
Flow chart delineating the derivation of the TBI cohort studied. TARN, Trauma Audit and Research Network; TBI, traumatic brain injury.

Figure 1 provides a summary flow chart of the numbers of patients in each cohort of the study audit, namely: those admitted directly to a neurosurgical centre (n=6358), those with a secondary admission (via a district general hospital, n=3682) to a neurosurgical centre and those not admitted to a neurosurgical centre (n=5880).

### Demographics and mechanism of injury

**Figure 2:**
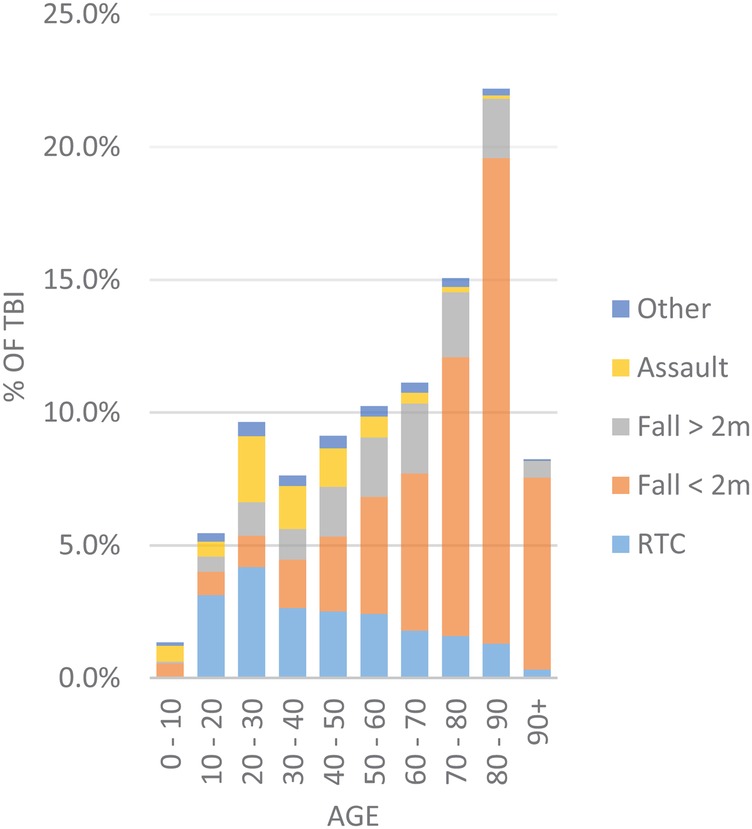
Proportion of all patients with TBI by age and mechanism of injury. RTI, road traffic collision; TBI, traumatic brain injury.

**Figure 3:**
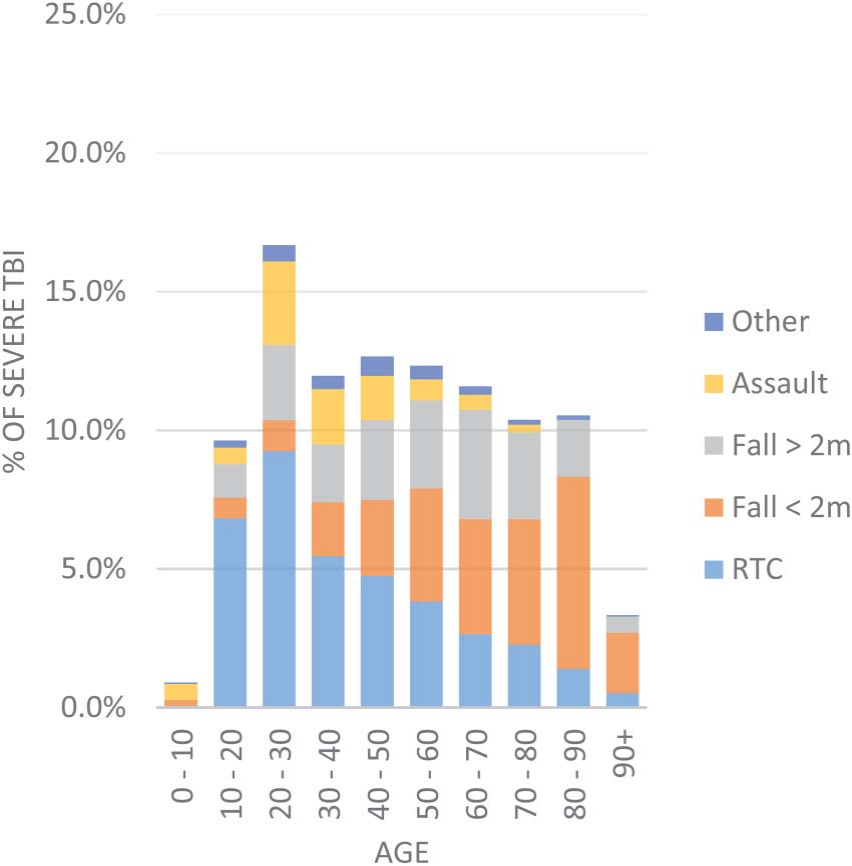
Proportion of patients with severe TBI (TBI in combination with GCS≤8) by age and mechanism of injury. RTI, road traffic collision.

For all TBI severities, there is a unimodal age distribution with a peak in those aged between 80 and 90, and this cohort represents more than one in five of those recorded as suffering from a TBI (figure 2). For those with severe TBI, there is a smaller peak between age 20 and 30 representing just over 15% of cases (figure 3). Younger patients are more likely to be injured as a result of road traffic collisions and assaults, while with increasing age, there is a concurrent increase in the proportion of patients injured following falls under 2 m. Of those patients with a documented admission GCS (n=15 080), the cohort is dominated by mild TBI (68%), with 26% with a severe TBI and only 6% with moderate TBI (table 1).

**Table 1:** Hospital transfer, airway management and length of stay

Category	Group	Severe TBI n (%)	Moderate TBI n (%)	Mild TBI n (%)	GCS not recorded n (%)	Total n (%*)*
Total number of patients		3915	899	10 266	742	15 822
Mode of arrival (direct admissions, n=13 824)	Ambulance	2504 (71.6%)	662 (83.8%)	6951 (76.6%)	51 (11.1%)	10 168 (73.5%)
	Car	3 (0.1%)	1 (0.1%)	65 (0.7%)	7 (1.5%)	76 (0.5%)
	Helicopter	660 (18.9%)	27 (3.4%)	309 (3.4%)	0 (0%)	996 (7.2%)
	Other	1 (0%)	1 (0.1%)	38 (0.4%)	1 (0.2%)	41 (0.3%)
	Unknown	329 (9.4%)	99 (12.5%)	1714 (18.9%)	402 (87.2%)	2544 (18.4%)
Transfer status (all patients), n=15 820	Direct admission to neurocentre	2353 (60.1%)	365 (40.6%)	3374 (32.9%)	167 (22.5%)	6259 (39.6%)
	Transfer into neurocentre	945 (24.1%)	181 (20.1%)	2298 (22.4%)	259 (34.9%)	3683 (23.3%)
	No neurocentre visit	617 (15.8%)	353 (39.3%)	4594 (44.7%)	316 (42.6%)	5880 (37.2%)
Hours to arrival at neurocentre (n=9940)	0–4	2225 (67.5%)	303 (55.5%)	2621 (46.2%)	46 (10.8%)	5195 (52.3%)
	4–12	438 (13.3%)	64 (11.7%)	733 (12.9%)	19 (4.5%)	1254 (12.6%)
	12–24	57 (1.7%)	21 (3.8%)	252 (4.4%)	8 (1.9%)	338 (3.4%)
	24–48	32 (1%)	9 (1.6%)	147 (2.6%)	6 (1.4%)	194 (2%)
	48–72	3 (0.1%)	1 (0.2%)	73 (1.3%)	1 (0.2%)	78 (0.8%)
	72+	23 (0.7%)	13 (2.4%)	249 (4.4%)	16 (3.8%)	301 (3%)
	Unknown	520 (15.8%)	135 (24.7%)	1597 (28.2%)	330 (77.5%)	2582 (26%)
Median length of stay (days) (IQR) n=15 820		12 (3–33)	11 (5–24)	9 (5–18)	10 (5–21)	9 (4–21)
Intubation location (direct admissions, n=13 824)	Prehospital	765 (21.9%)	0 (0%)	0 (0%)	0 (0%)	765 (5.5%)
	ED	2236 (63.9%)	0 (0%)	0 (0%)	0 (0%)	2236 (16.2%)
	Critical care	142 (4.1%)	71 (9%)	257 (2.8%)	21 (4.6%)	491 (3.6%)
	Ward	0 (0%)	0 (0%)	4 (0%)	2 (0.4%)	6 (0%)
	Not intubated	354 (10.1%)	719 (91%)	8815 (97.1%)	438 (95%)	10 326 (74.7%)

ED, emergency department; GCS, Glasgow Coma Score; TBI, traumatic brain injury.

### Transfer to hospital and airway management

Table 1 summarises data on hospital transfers and airway management stratified by severity of injury of TBI. The most common mode of transport to hospital is ambulance (74% overall). Seven per cent of patients are recorded as being transported by helicopter, although in patients with severe TBI, this increases to 19%. Direct admission to a neurosurgical centre from the scene of injury occurred in ∼40% of patients overall and over 60% of patients with severe TBI. This proportion was lower for patients with moderate and mild TBI (41% and 33%) but significant proportions were subsequently transferred (20% and 22%, respectively).

Over 80% of patients with severe TBI were admitted to a neurosurgical centre within 12 hours of injury with 68% within 4 hours. Eighty-six per cent of patients presenting with a severe TBI had definitive airway management (defined as endotracheal intubation, tracheostomy or cricothyroidotomy) prehospital or in the ED. Definitive airway management was rarely required for patients with less severe injuries.

### Time to intervention

Table 2 summarises the data on the time intervals from injury and admission to investigation and intervention. In those patients admitted directly from the scene of injury with a GCS≤8, a median of 0.5 hours was taken to image with CT scan. Median time from arrival to imaging was 0.9 hours for moderate TBI and 1.7 hours for mild injuries. The median time taken from admission to craniotomy was 2.6 hours for severe TBI and 8.6 hours for moderate TBI. If the time to craniotomy, in severe TBI, is calculated from the time of the incident, this increases to 4 hours for direct transfers to a neurosurgical centre and 7.3 hours for those who required a secondary transfer. Median time to intracranial pressure (ICP) monitoring following admission to a neurosurgical centre was 3.1 hours following severe TBI. Smaller numbers of patients with mild or moderate TBI required craniotomy (3.1% and 2.7%, respectively) or ICP monitoring (0.7% and 2.1%, respectively), and in general, this was performed within 24 hours of arriving in hospital.

**Table 2:** Median time to CT scanning, craniotomy and ICP monitoring from hospital arrival/incident*

TBI severity	Measured from	Category	n†	Median hours	IQR lower bound	IQR upper bound
Severe TBI	Arrival time	CT	3307	0.5	0.3	0.8
		Craniotomy	457	2.6	1.6	10.1
		ICP monitoring	411	3.1	1.8	7.4
	Incident time	CT	3565	2.0	1.5	3.2
		Craniotomy (direct)	423	4.0	2.9	17.2
		Craniotomy (transfer)	262	7.3	5.3	19.0
		ICP monitoring	571	5.8	3.5	11.3
Moderate TBI	Arrival time	CT	766	0.9	0.5	1.9
		Craniotomy	45	8.6	2.8	47.9
		ICP monitoring	16	8.4	2.7	47.9
	Incident time	CT	751	2.5	1.8	5.5
		Craniotomy (direct)	42	15.8	5.3	65.8
		Craniotomy (transfer)	24	38.3	9.7	226.0
		ICP monitoring	19	8.6	6.1	48.8
Mild TBI	Arrival time	CT	8740	1.7	0.7	3.3
		Craniotomy	218	19.2	6.6	97.3
		ICP monitoring	41	11.6	5.8	28.6
	Incident time	CT	8173	3.7	2.3	8.6
		Craniotomy (direct)	170	21.4	7.4	119.2
		Craniotomy (transfer)	320	53.2	16.3	240.6
		ICP monitoring	69	18.3	8.4	38.0
GCS not recorded	Arrival time	CT	367	3.5	1.4	16.5
		Craniotomy	14	15.4	8.5	36.8
		ICP monitoring	2	10.1	0.0	0.0
	Incident time	CT	202	8.5	2.4	36.6
		Craniotomy (direct)	4	189.1	0.0	0.0
		Craniotomy (transfer)	19	40.0	12.4	560.3
		ICP monitoring	7	11.3	10.0	11.7

*Hospital arrival time is recorded in almost 100% of cases; incident time is recorded in ∼75% of cases. Intervals measured from incident time include patients that are transferred between hospitals; those measured from arrival time only include patients admitted directly from the scene of injury.

†n values relate to the number of observations in each cohort. For example, 3307 patients with a severe TBI underwent CT scanning and have their arrival and CT scan dates and times recorded.

GCS, Glasgow Coma Score; TBI, traumatic brain injury.

### Complications in hospital

Table 3 summarises the documented complications following TBI. Overall, over 19% of patients are recorded as suffering a complication, and in the severe TBI cohort, this incidence increases to almost 30%. There is a wide range of complications; the most frequent in the severe TBI cohort were bronchopneumonia (4.9%), in-hospital seizure (2.9%), sepsis (3.1%) and pleural effusion (2%). These were also the most common complications in the cohort as a whole.

**Table 3:** Inpatient complications stratified by severity of injury

Complication	Severe TBI	Moderate TBI	Mild TBI	GCS not recorded	Total
Aspiration	63 (1.5%)	8 (0.9%)	48 (0.5%)	5 (0.6%)	124 (0.8%)
Bronchopneumonia	203 (4.9%)	32 (3.4%)	209 (2%)	17 (2.2%)	461 (2.8%)
Pleural effusion	84 (2%)	9 (1%)	68 (0.7%)	12 (1.6%)	173 (1.1%)
Seizure in hospital	119 (2.9%)	22 (2.4%)	141 (1.4%)	15 (1.9%)	297 (1.8%)
Sepsis	129 (3.1%)	9 (1%)	107 (1%)	13 (1.7%)	258 (1.6%)
Other	624 (15%)	106 (11.4%)	836 (8%)	88 (11.4%)	1654 (10.1%)
No complications recorded	2944 (70.7%)	744 (80%)	9027 (86.5%)	623 (80.6%)	13 338 (81.8%)

GCS, Glasgow Coma Score; TBI, traumatic brain injury.

### Risk-adjusted outcomes at neurosurgical units

**Figure 4:**
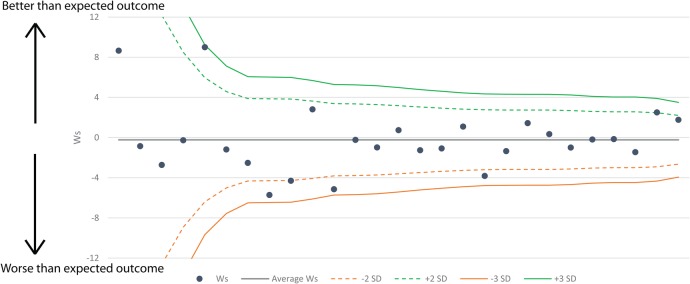
Funnel plot showing the Ws^n^ for neurosurgical units in England and Wales between April 2014 and June 2015. Ws^n^, W scores derived using the Ps14 multivariate logistic regression model.

Figure 4 shows a funnel plot^12^ of the risk-adjusted W scores derived using the Ps14^n^ model (Ws^n^) for each unit on the y-axis against a precision (1/SE) based rank on the x-axis. A positive Ws^n^ indicates that a site is performing better than the model predicts, a negative value indicates worse performance. The ‘funnel’ refers to the 2 and 3 SD lines, plotted around the mean Ws that narrow as the precision increases. All units are within the 3 SD lines and most units fall within the 2 SD lines; four units are outside the **−**2 SD line and two units are above the +2 line. The Ws^n^ value for a given site relative to the position of the SD lines indicates if their performance significantly differs from that of their peers.

## Discussion

This audit incorporates prospectively collected data on a large number of patients, including from every neurosurgical unit in England and Wales, and provides the most comprehensive and up to date report of outcomes following TBI in England and Wales.

### Demographics and mechanism of injury

The cohort of patients in the TARN database mimics data from other large TBI databases and the demographics and mechanism of injury closely mirror those from other series of patients with TBI in the developed world.^13–15^ The most common injuries are those in elderly people following trips and falls, while in younger patients, the most common causes are road traffic collisions and assault and these are more likely to present as severe TBI. We have provided a breakdown of delay to transfer to neurosurgical centre and complication rates by 10- year age bracket in the online supplementary information. This demonstrates that despite comparable transfer times between adult groups, there are a smaller number of children aged 0–10 years transferred within 4 hours (32%) as compared with adult age brackets (range 45–61%). This does not lead to an increased frequency of complications and we speculate that this is due to specialised transfer team involvement for young children (Children's Acute Transfer Service, CATS). Interestingly, only 6% of patients with TBI fall into the moderate (GCS 9–13) category calling into question whether the current GCS thresholds for severity accurately reflect the underlying condition: intuitively, one might expect that more severe injuries are increasingly rare. Other epidemiological studies in high-income countries reinforce this pattern of falls as a common aetiology in elderly patients.^15^

### Transfer to hospital

While the majority of patients are transported to hospital by land ambulance, there is an increasing use of helicopter ambulance for those patients with severe TBI. These patients are increasingly being transported directly to major trauma centres (MTCs) as part of the National Health Service (NHS) plan to centralise the management of complex trauma. The choice of mode of transport to hospital and choice of local hospital versus a neurosurgical or MTC is a complex one. Factors such as the physiological stability of a patient on scene and the geography of local emergency services dictates individualisation of decision-making and it is difficult to mandate transport of a group of patients to a given location. A recent publication from the TARN registry^16^ found no association between the duration of the prehospital interval and deteriorating physiological parameters. We did not find a difference in complication rate between these two cohorts (see online supplementary information). There are also challenges with the reliable identification of TBI in the prehospital environment and current strategies suffer from significant undertriage and overtriage rates making secondary transfer into neurosurgery, a necessary pathway for some patients with TBI.^17^ However, in patients with severe TBI, who are likely to survive and require treatment, we would expect transfer to a neurosurgical centre once physiological stability has been achieved.^18^ This is supported by NICE guidance—in our series 84% of patients with severe TBI received neuroscience care, suggesting reasonable adherence. For mild and moderate TBI, an individual decision is required as to the need and rapidity of transfer to a neurosurgical centre. In a resource-limited environment however, an efficient use of specialist beds necessitates some degree of triage at local centres before transfer to a specialist centre.

### Time to intervention

The median and upper quartile time to CT is within the 1-hour from ED arrival target defined by the NICE head injury guidelines^18^ for patients at high risk of TBI requiring neurosurgery (GCS<13=moderate/severe TBI); NICE recommends CT brain scan for GCS 15 patients with additional risk factors but not high risk, within 8 hours of injury. Patients with mild TBI with GCS 13–14 on arrival at hospital should have CT within an hour if the GCS does not reach 15 within 2 hours of injury. Sequential ED GCS readings are not well recorded on TARN but table 2 suggests that this NICE recommendation also has reasonable adherence. The Brain Trauma Foundation surgical guidelines^19^ recommend that acute intracranial haemorrhages are treated as quickly as possible in those patients presenting in coma. The evidence for rapid treatment by craniotomy is strongest in those presenting with a fixed, dilated pupil.^20^ In this regard, our data show direct transfer to a neurosurgical centre facilitates more rapid surgery and as such we support current ambulance service trauma triage guidelines that direct primary transportation from scene to a neurosurgical centre for patients with a unilateral fixed, dilated pupil in the context of severe TBI and a patent airway.^17^ Consideration should also be given to establishing guidelines for direct transfer of other patients with TBI from the scene to neurosurgical units, notwithstanding the difficulty in accurate identification of patients in the prehospital setting, and refining referral mechanisms from district hospitals/trauma units to MTCs with neurosurgical capability. Any guidelines must reflect the low requirement for craniotomy and ICP monitoring in mild (3.1% and 0.7%) and moderate (2.7% and 2.1%) TBI, such that in the majority of these patients expedited transfer to a neurosurgical centre may be unnecessary.

### Complications and risk-adjusted outcomes at neurosurgical units

Patients with TBI are susceptible to a wide range of complications as evidenced by the reported complications. Respiratory complications predominate as would be expected in critically ill patients with a reduced conscious state or those in an intensive care environment. The analysis shows that five units lie outside of the 2 SD control limits; however, they and all other units are within the 3 SD limits. A single centre is close to the positive 3 SD limit, but this is one of the units with lower precision where we expect to see larger variation from the mean. As such these data suggest that there are no outlying units in terms of risk-adjusted mortality in neurosurgical care for patients suffering from TBI in England and Wales. Further studies are required to address the quality of survival in terms of outcome beyond mortality. On the basis of the funnel plot, it appears that there is a slight excess of units falling below the expected standardised mortality ratio (worse than expected outcome). This is most likely due to the expected (average) value being skewed upwards by the two centres with low precision and very high Ws^n^ scores. In addition, a significant proportion of the centres below the expected value are those with lower precision, the higher precision units on the right side of the plot are more evenly balanced.

### Study limitations

Although this audit is comprehensive, there are certain limitations to using aggregate data of this type. First, as with many studies that use GCS, we have used a composite score rather than the individual components, despite each component of the GCS being on a categorical scale. This is partly addressed by the validation of this approach by the IMPACT model.^10^ ^21^ Second, there is some variability in the reporting of GCS, such that ‘first’ GCS is sometimes used interchangeably with ‘postresuscitation’ GCS.^21^ Third, we have not addressed the decision-making with regard to transfer of patients from peripheral to neurosurgical centres, and the possibility of regional variation. This could potentially have an effect on TBI survival rates in specialist centres if there is a variation in transfer criteria, particularly for older patients who may have poorer prognosis.^22^ Finally, there is some variability in patient recruitment into the TARN database, over the time period of the study neuroscience centres recruited almost 100% of relevant patients, outside of these hospitals, however the average is roughly 65%. Nevertheless, we hope by compiling data on more than 15 000 patients, we are able to provide robust data on TBI management in England and Wales.

## Conclusion

This report provides the first England and Wales audit of its type with a large number of patients that is commensurate with the largest cohorts of patients currently published in TBI, namely the CRASH and IMPACT studies. This provides a robust baseline for further comparisons of outcomes in a transparent and reproducible fashion. The data we present confirm that England and Wales trauma management broadly meets the NICE guidelines and achieves a consistent standard across all regions and neurosurgical units. The NICE guidelines are broad and rightly err on the side of caution in the necessity for CT imaging and discussion with specialist centres.^18^ Specifically, they are for the management of head injury, rather than traumatic brain injury, and the recommendations address CT imaging and appropriate transfer to neurosurgical centres, rather than ICP monitoring and the need for craniotomy, although this is a possibility in the future. The need for these guidelines to be used in a range of ED settings necessitates this approach, although data presented here highlight that neurosurgical intervention is rarely required for those presenting with mild or moderate TBI. The increasing need for public engagement with regard to surgical outcomes, and the related political imperative to provide this within the NHS will become the *status quo*.
