# Supplementary material for: Traumatic brain injury in England and Wales: prospective audit of epidemiology, complications and standardised mortality
Source: BMJ Open. 2016 Nov 24;6(11):e012197. doi: 10.1136/bmjopen-2016-012197 (PMC5168492; doi:10.1136/bmjopen-2016-012197)
Supplement: supplementary data [file bmjopen-2016-012197supp.pdf]

Appendix

The Ps14^n model is a modification of the Ps14 model as described by Bouamra in 2015 with the addition of pupil reactivity. It has been derived from 39451 patients recorded in the TARN database with head injuries of AIS 3 or greater severity admitted to hospital between 2012 and 2015. Table 1 describes the characteristics of these patients and table 2 contains the regression coefficients of which the prediction model is formed.

The model is shown to have good discriminant power (AuROC 0.882, 95% CI 0.878 to 0.887). The calibration of the model was assessed using the Brier score, this shows how close predictions are to the actual outcome. Its value varies between 0 (perfect) and 0.25 (useless), the model shows a value of 0.086. The value for the HL-statistic (Hosper & Lemeshow chi^2(8)) is 19.51, P-value 0.0124 (as the p-value is <0.05 the model failed the goodness-of-fit test but this is expected with the HL-test as it is too sensitive to large sample sizes). Figure 1 shows a graphical assessment of calibration using 200 bootstrap simulations and shows an almost perfect calibration: the observed and expected are aligned to the 45° line.

Table 1. Characteristics of the patients

**n**	39451
**Age**	
Median (IQR)	62.6 (35.8 - 81.4)
**ISS**	
Median (IQR)	24 (16 - 26)
**GCS**	
Median (IQR)	14 (11 - 15)
**Gender**	
Female	13927 (35.3%)
Male	25524 (64.7%)
**Comorbidity**	
No comorbidity	19776 (50.1%)
1 - 5	10617 (26.9%)
6 - 10	3417 (8.7%)
> 10	1585 (4.0%)
Not recorded	4056 (10.3%)
**Mortality**	16.0%

Table 2. Coefficients of the model

Variables	Regression Coefficients	p-value	Odds ratio 95% CI	
$(10/ISS)^2$ - 0.1920	3.3294	<0.0001	22.9	33.8
$(10/ISS)^2 * \log_e(ISS/10)$ - 0.1584	8.2092	<0.0001	1719.9	7785.4
**GCS**				
GCS =3	-3.0652	<0.0001	0.04	0.05
GCS 4 -5	-2.6485	<0.0001	0.06	0.08
GCS 6 -8	-1.8352	<0.0001	0.14	0.18
GCS 9 -12	-1.3348	<0.0001	0.23	0.30
GCS 13 - 14	-0.4704	<0.0001	0.57	0.69
GCS 15 (reference)	0.0000		1	1
Intubated	-2.5212	<0.0001	0.06	0.11
**Charlson Index**				
0 (reference)	0.0000		1	1
1 - 5	-0.4593	<0.0001	0.58	0.69
6 - 10	-0.7754	<0.0001	0.41	0.52
>10	-1.1841	<0.0001	0.26	0.36
Not recorded	-0.6575	<0.0001	0.46	0.58
**Age**				
0 - 5	-0.0236	0.92	0.63	1.51
6 - 10	0.5668	0.07	0.95	3.25
11 - 15	0.0356	0.87	0.67	1.60
16 - 44 (reference)	0.0000		1	1
45 - 54	-0.5065	<0.0001	0.51	0.72
55 - 64	-1.0091	<0.0001	0.31	0.44
65 - 75	-1.6125	<0.0001	0.17	0.23
>75	-2.7684	<0.0001	0.05	0.07
**Gender**				
Male (reference)	0.0000		1	1
Female	-0.0216	0.84	0.79	1.21
**Age by gender interaction**				
0 - 5 & Female	-0.1968	0.55	0.43	1.58
6 - 10 & Female	-0.3357	0.55	0.24	2.14
11 - 15 & Female	0.8354	0.11	0.83	6.49
45 - 54 & Female	-0.2911	0.11	0.52	1.07
55 - 64 & Female	0.2989	0.09	0.95	1.92
65 - 75 & Female	-0.0281	0.85	0.73	1.30
>75 & Female	0.1084	0.36	0.89	1.40
**Pupil reactivity**				
Both reactive (reference)	0.0000		1	1
Abnormal (both reactive)	-0.4383	<0.0001	0.57	0.73
Abnormal (1 reactive)	-0.5325	<0.0001	0.50	0.69
Neither reactive	-2.0874	<0.0001	0.11	0.14
**Constant**	4.9208	<0.0001	119.10	157.59

Figure 1: Model Calibration

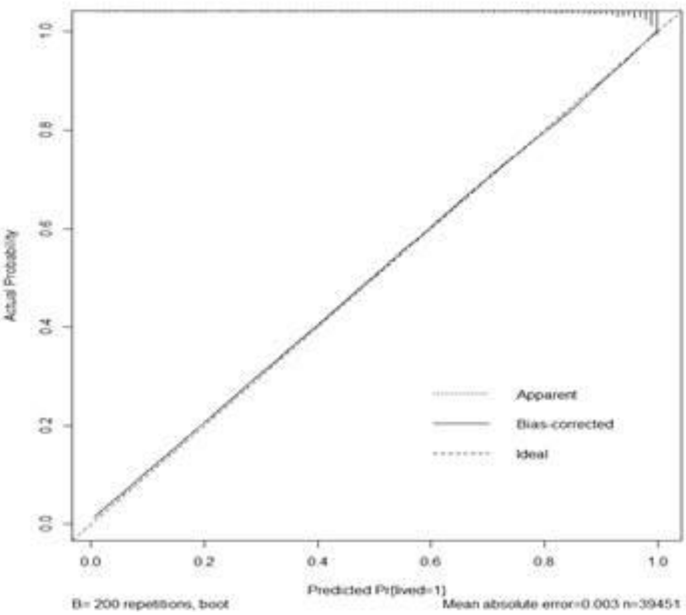

Table 3: Hours to arrival at neurosurgical centre stratified by age

Hours to arrival at neurocentre	Age									
	0 - 10	10 - 20	20 - 30	30 - 40	40 - 50	50 - 60	60 - 70	70 - 80	80 - 90	90+
**0 - 4**	50 (31.8%)	434 (60.5%)	748 (60.1%)	543 (55.9%)	550 (49.5%)	603 (50.2%)	591 (49.5%)	604 (44.7%)	799 (51.4%)	293 (58.8%)
**4 - 12**	6 (3.8%)	8 (1.1%)	25 (2%)	6 (0.6%)	28 (2.5%)	31 (2.6%)	27 (2.3%)	19 (1.4%)	39 (2.5%)	8 (1.6%)
**12 - 24**	31 (19.7%)	112 (15.6%)	162 (13%)	132 (13.6%)	159 (14.3%)	190 (15.8%)	165 (13.8%)	155 (11.5%)	125 (8%)	47 (9.4%)
**24 - 48**	11 (7%)	19 (2.6%)	35 (2.8%)	35 (3.6%)	41 (3.7%)	56 (4.7%)	46 (3.8%)	51 (3.8%)	43 (2.8%)	9 (1.8%)
**48 - 72**	1 (0.6%)	7 (1%)	8 (0.6%)	8 (0.8%)	10 (0.9%)	11 (0.9%)	9 (0.8%)	9 (0.7%)	14 (0.9%)	2 (0.4%)
**72+**	6 (3.8%)	6 (0.8%)	13 (1%)	10 (1%)	28 (2.5%)	28 (2.3%)	34 (2.8%)	53 (3.9%)	94 (6.1%)	27 (5.4%)
**Unknown**	52 (33.1%)	131 (18.3%)	254 (20.4%)	237 (24.4%)	294 (26.5%)	283 (23.5%)	323 (27%)	459 (34%)	439 (28.3%)	112 (22.5%)

Table 4: Rate of complications by age and transfer status

Age	n	Complications	% with complications
**0-10**	211	35	16.6%
**10-20**	863	100	11.6%
**20-30**	1523	184	12.1%
**30-40**	1206	189	15.7%
**40-50**	1443	197	13.7%
**50-60**	1620	271	16.7%
**60-70**	1760	277	15.7%
**70-80**	2380	395	16.6%
**80-90**	3508	613	17.5%
**90+**	1305	221	16.9%
**Total**	**15819**	**2482**	**15.7%**

Age	n	Complications	% with complications
No transfer	10893	1611	14.8%
Transfer	4926	871	17.7%
**Total**	**15819**	**2482**	**15.7%**

Table 5: Mortality stratified by GCS and Head AIS

TBI severity	n	Mortality			Predicted mortality %
		n	%	95% CI	
**Mild TBI**	9285	730	7.9%	7.3% - 8.4%	8.6%
**Moderate TBI**	790	178	22.5%	19.6% - 25.4%	23.6%
**Severe TBI**	3228	1304	40.4%	38.7% - 42.1%	36.1%*
**GCS not recorded**	629	98	15.6%	12.7% - 18.4%	12.4%
**Total**	**13932**	**2310**	**16.6%**	**16% - 17.2%**	**16.0%**

AIS TBI severity	n	Mortality			Predicted mortality %
		n	%	95% CI	
**3**	2480	155	6.3%	5.3% - 7.2%	5.9%
**4**	5828	475	8.2%	7.4% - 8.9%	9.6%
**5**	5614	1671	29.8%	28.6% - 31%	26.9%
**6**	10	9	90.0%	71.4% - 100%	68.0%
**Total**	**13932**	**2310**	**16.6%**	**16% - 17.2%**	**16.0%**

Outcome	n	Outcome at discharge	Outcome at 30 days
**Alive**	11622	9642	1980
**Dead**	2310	2310	0
**Total**	**13932**	**11952**	**1980**

*Although in this cohort of severe TBI patients predicted mortality differs from actual mortality, the model is not specifically calibrated by the severity subsets. Overall, mortality across all patients ('total' row) matches the predicted mortality from the PS14[n] model.

Table 6: Complication rate stratified by location of transfer

Transfer status	n	Complications	% with complications
No transfer	10893	1611	14.8%
Transfer	4926	871	17.7%
**Total**	**15819**	**2482**	**15.7%**
